# A Young man with Delirium Tremens, Pellagra and Alcoholic neuropathy. Case report and review of pharmacological treatment

**DOI:** 10.1192/j.eurpsy.2022.2134

**Published:** 2022-09-01

**Authors:** B. Díez Valle, P. Coucheiro Limeres

**Affiliations:** 1 Hospital Universitario Severo Ochoa, Psychiatry, Leganés, Spain; 2 Hospital Universitario José Germain, Psychiatry Department, Leganés, Spain

**Keywords:** alcohol withdrawal, antiepileptics, alcoholism, pharmacological treatment

## Abstract

**Introduction:**

We present the case of a 36-year-old male with chronic alcoholism who suffered Delirium Tremens and other complications during hospital admission and who recovered thanks to treatment with benzodiazepines and antiepileptics using a cross tapering strategy.

**Objectives:**

Presentation of a case and review of the available literature on the pharmacological treatment of alcohol withdrawal.

**Methods:**

A 36-year-old man was hospitalised for extensive dermatological lesions suggestive of Pellagra. He acknowledged a daily consumption of six litres of beer, was homeless and had a poor and unvaried diet. After 48 hours, the patient began to present hyperreflexia, disorientation and delusions of harm and was diagnosed with Delirium Tremens.

**Results:**

The case was managed jointly by Internal Medicine and Psychiatry. High doses of Chlorazepate (up to 400 mg daily), Tiapride (up to 600 mg daily) and Thiamine (300 mg daily) were prescribed. After 5 days of treatment, the patient started to improve but severe pain appeared in the lower limbs suggestive of alcoholic neuropathy. Gradually the treatment was replaced by Pregabalin (up to a dose of 1200 mg daily) which was effective in calming the late withdrawal and partially controlling the lower limb pain.

**Conclusions:**

Benzodiazepines remain the first-line agent for severe withdrawal, while some antiepileptic drugs have proven useful in mild-moderate withdrawal and relapse prevention. Switching to antiepileptic drugs during follow-up should be considered because of the lower risk of dependence and respiratory depression, as well as the positive effects on the “kindling” phenomenon.

**Disclosure:**

No significant relationships.

